# Attenuated Total Reflectance Fourier Transform Infrared Spectroscopy: An analytical technique to understand therapeutic responses at the molecular level

**DOI:** 10.1038/srep16649

**Published:** 2015-11-16

**Authors:** Sushma Kalmodia, Sowmya Parameswaran, Wenrong Yang, Colin J. Barrow, Subramanian Krishnakumar

**Affiliations:** 1 Department of Nano biotechnology, Vision Research Foundation, Sankara Nethralaya, 18, College Road, Nungambakkam, Chennai – 600 006, India; 2 Centre for Chemistry and Biotechnology, Deakin University, Geelong campus, VIC 3216, Australia; 3 Radheshyam Kanoi Stem Cell laboratory, Vision Research Foundation, Sankara Nethralaya, 18, College Road, Nungambakkam, Chennai – 600 006, India

## Abstract

Rapid monitoring of the response to treatment in cancer patients is essential to predict the outcome of the therapeutic regimen early in the course of the treatment. The conventional methods are laborious, time-consuming, subjective and lack the ability to study different biomolecules and their interactions, simultaneously. Since; mechanisms of cancer and its response to therapy is dependent on molecular interactions and not on single biomolecules, an assay capable of studying molecular interactions as a whole, is preferred. Fourier Transform Infrared (FTIR) spectroscopy has become a popular technique in the field of cancer therapy with an ability to elucidate molecular interactions. The aim of this study, was to explore the utility of the FTIR technique along with multivariate analysis to understand whether the method has the resolution to identify the differences in the mechanism of therapeutic response. Towards achieving the aim, we utilized the mouse xenograft model of retinoblastoma and nanoparticle mediated targeted therapy. The results indicate that the mechanism underlying the response differed between the treated and untreated group which can be elucidated by unique spectral signatures generated by each group. The study establishes the efficiency of non-invasive, label-free and rapid FTIR method in assessing the interactions of nanoparticles with cellular macromolecules towards monitoring the response to cancer therapeutics.

Over the last few decades, Fourier Transform Infrared (FTIR) spectroscopy has become a popular spectroscopic technique in the field of cancer diagnosis^1^,^2^,^3^. This has opened a new avenue in the field of molecular diagnosis which can effectively identify the presence or absence of specific interaction between the biomolecules in the cellular component, qualitatively and quantitatively^4^. FTIR micro spectroscopy is an approach that has advantages over some of the available molecular biological and histopathological techniques which rely on statistical confidence and operator expertise^5^.

The techniques that are routinely used in diagnosing cancer in tissue sections include, immunohistochemistry (IHC), Fluorescent *in-situ* Hybridization (FISH) and Chromogenic *in situ* hybridization (CISH)^6^. Although, these techniques are extensively used for diagnosis, there is considerable subjectivity in the interpretation of the results owing to the inter and intra -observer errors^7^. The success of the *in-situ* hybridization and immunohistochemical techniques including tissue microarray rely on several physical and chemical aspects such as specificity and sensitivity of the probes and antibodies, hybridization/incubation parameters, chemical reagents used for tissue processing, labeling and detection^6^,^7^.

In addition, these diagnostic methods do not aid in studying the interaction of different biomolecules such as proteins, lipids, nucleic acids in a given sample. For instance, *in-situ* hybridization is limited to studying the nucleic acids while, immunohistochemistry aids in understanding the protein expression. Additionally, multiple assays are required to identify multiple protein antigens or nucleic acid targets. However, the origin of cancer, its progression and its response to therapy is dependent on the molecular interactions occurring between biomolecules (lipids-nucleic acids, protein-lipids; protein-nucleic acid) and not on single biomolecule. A technique that has the capacity to assess the cumulative molecular interactions irrespective of the presence or absence of specific target molecule might be more effective. Hence, we hypothesized that spectroscopic methods would be helpful in the understanding of the molecular interaction with therapeutic molecules. The vibrational spectroscopy relies on the non-perturbing identification of molecules arising from the inherent chemical composition of the tissue sample^8^. Spectroscopic methods such as FTIR offers a rapid and non-invasive approach of analyzing biomolecules by generating distinct and unique spectral signatures derived from various endogenous functional groups present in the biomolecules^9^,^10^,^11^ FTIR is a reagent-free technique with minimal processing and requires less amount of sample to analyze the biochemical finger print. In addition, FTIR can also be adapted for clinical applications in place of other techniques that use radiations such as X-rays and gamma rays which are destructive for the biological samples.

In this study, we explored the utility of Attenuated Total Reflectance Fourier Transform Infrared spectroscopy (ATR-FTIR) technique to understand whether the method has the capacity to distinguish the mechanism involved in therapeutic response using nanoparticle mediated targeted therapy and retinoblastoma (RB) xenograft mouse model. Nanoparticle based targeted therapy is preferred for this study for the following reasons: (i) Several nanocarrier based approaches have emerged for cancer therapy^12^,^13^,^14^(ii) Almost, all the studies involving nanocarrier based approaches have utilized tumor growth reduction as the end point and do not decipher the mechanism of action (iii) None of the studies utilizing nanocarrier based approaches have explored the interaction of the nanoparticles with biomolecules to our knowledge; (iv) There limited information reported on the utility of ATR-FTIR in studying the interactions of nanoparticles with the biomolecules in cancer therapy^13^,^14^.Towards achieving the aim, we generated xenografts of retinoblastoma in nude mice and subjected them to treatment with either GNPs-1 (Gold nanoparticles prepared using *Vitis vinifera* extract, as the reducing agent) functionalized with HDM2 peptide or GNPs-2 (Gold nanoparticle prepared using Sodium citrate, as the reducing agent) functionalized with HDM2 peptide. The effectiveness of both the GNP conjugates in the tumor reduction was assessed. We have applied ATR-FTIR technique to assess the interactions of the GNPs-conjugates with the cellular macromolecules such as proteins, lipids, and nucleic acids in the tumor tissue.

The results indicate an interaction of the novel conjugate (GNPs-HDM2) with the tumor, causing a change in the chemical composition, which is reflected as a difference in the spectroscopic signatures as revealed by the ATR-FTIR method. The study confirms the utility of ATR-FTIR to investigate the interaction of the GNPs-HDM2 with cellular component at the molecular level. The study provides a ‘proof-of-principle’ for evaluating the efficiency of non-invasive, label-free and rapid ATR-FTIR method in assessing the interactions of nanoparticles with cellular macromolecules towards establishing the outcome of cancer therapeutics.

## Methods

### Generation of retinoblastoma (RB) xenograft model

**Figure 1:**
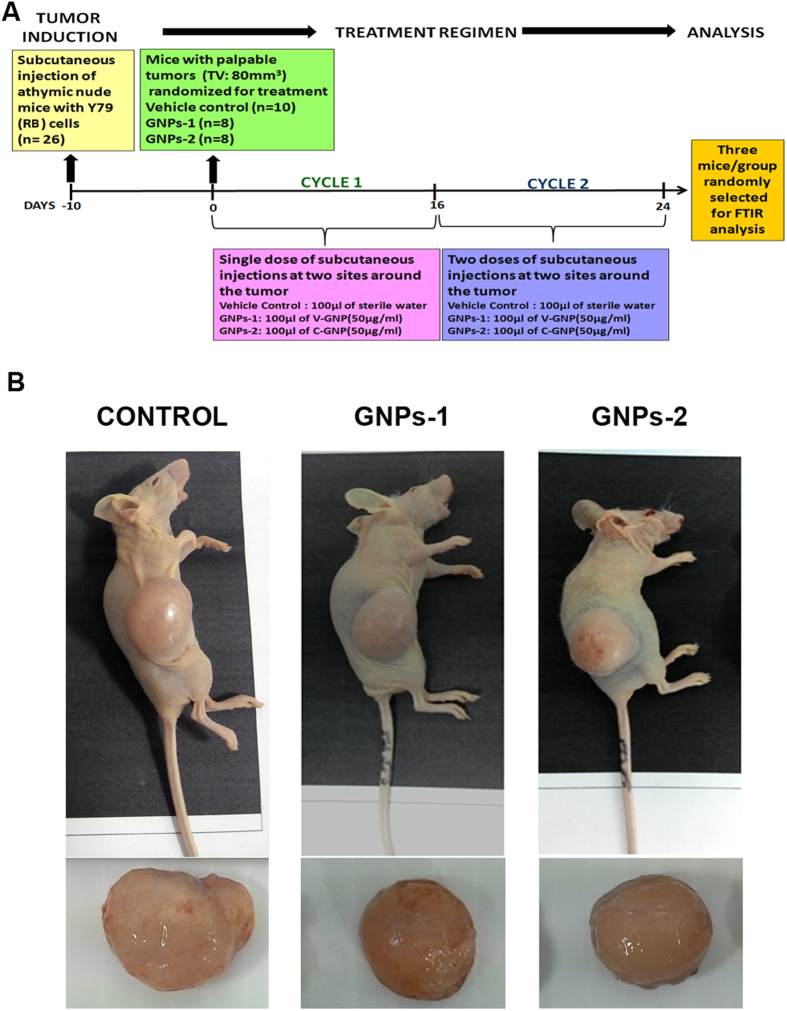
Treatment of RB xenograft models with GNP conjugates and harvesting of tumors for ATR-FTIR analysis. A schematic representation of generation of RB xenograft model; the treatment regimen using GNP conjugates and harvesting of tissue for ATR-FTIR analysis is provided (**A**). Representative images of Hsd: Athymic Nude-Foxn1^nu^ mice with RB xenograft 24 days post-treatment of control, GNPs-1, GNPs 2 and their respective tumor harvested for FTIR analysis (**B**).

All the methods were carried out in accordance with approved institutional ethical guidelines (institutional ethics study code no: 383-2013-P; SLAR/ONC/SAN-002). The animal experiment protocols were approved by the “Committee for the Purpose of Control and Supervision of Experiments on Animals (CPCSEA)”. For the generation of xenograft, second passage of human RB (Y79) cell line obtained from ATCC, USA at a cell density of 10 × 10^6^ cells in 200 μl of serum free media containing 50% of matrigel was subcutaneously injected in the flanks/back of nude mice (Hsd: Athymic Nude-Foxn1^nu^). The implanted area was monitored for tumor growth daily till the tumor attained palpable size with a tumor volume (TV ≈ 80 mm^3^). The animals were randomized into 3 groups; control (n = 10), GNPs-1(n = 8) and GNPs-2 (n = 8) (Fig. 1).

### Preparation of GNPs-1 and GNPs-2 conjugates and their characterization

The GNPs-1 and GNPs-2 were prepared as described previously^15^,^16^. The GNPs- anti HDM2 peptide conjugates were prepared by mixing varying concentrations of 3-mpa (Mercaptopropionic acid) modified anti-HDM2 peptide with purified GNPs (GNPS-1 and GNPs-2, separately). The mPEG-thiol stabilized GNPs were prepared by mixing 50 μM (atom of Gold) of GNPs with 1 mg of MPEG-SH-1000. The solution was stirred for 12 h followed by removal of un-reacted PEG by centrifugation. The PEG stabilized GNPs were mixed with 250 μM of HDM2 peptide and mixed in a rotary platform for 24 h. The final conjugates were subjected to washing and characterized by FTIR and TEM (Supplementary methods). The conjugates were re-suspended in distilled water to obtain a final stock concentration of (50 μg/ml) for further experimentation.

### Treatment of RB xenograft models with GNP conjugates

The animals were grouped as control, GNPs-1 treated and GNPs-2 treated. The control, GNPs-1 and GNPs-2 animals were subcutaneously injected with 100 μl of sterile water; GNPs-1(50 μg/ml) and GNPs-2 (50 μg/ml) respectively. The injection site was kept uniform in all the animals in all groups and was made subcutaneously at two sites around the tumor. The injection was provided in two-cycles, the first cycle was from 0 till 16 days with a single dose per day and the second cycle was from 17th till 24th day with two doses/day. The period of treatment was fixed as 24 days based on the tumor doubling time (approximately 5 days) and the fact that the total tumor volume in the control does not exceed 2000 mm^3^ (for ethical reasons). The tumor growth and the body weight of the mice were monitored every three days. The tumor volume was measured with a caliper and derived by the formula: Tumor Volume (TV) (mm3) = L × W^2^/2); where L is the length and W is the width. The relative tumor volume (RTV) was calculated for each group using the formula: TV^n^/TV^0^ where TV^n^ is the tumor volume on a given day and TV^0^ is the volume on day 0. The mean ± SEM were calculated for all the groups and Two way ANOVA was used for calculating statistical significance. The tumor growth inhibition (TGI) was calculated using the formula: (1 − T/C) × 100 (where T is the mean TV of the test group on day n – mean TV of the test group on day 0 and C is the mean TV of the control on day n – mean TV of the control group on day 0). Two way ANOVA was used for calculation of statistical significance and p < 0.05 was considered as significant. After completion of the experiment period, the animals were sacrificed and tumors were collected (Fig. 1). The subcutaneous tumor tissue was snap frozen for further analysis. The dose tolerance of the conjugates was assessed by monitoring body weight, biochemical analysis for liver function (alanine aminotransferase (ALT) and aspartate aminotransferase (AST)), kidney function (Blood urea nitrogen(BUN) and Creatinine), differential leucocyte counts (DLC) and histopathological analysis of vital organs. The tissue was utilized for real- time PCR, apoptosis array analysis (Supplementary methods) and FTIR analysis.

### Processing of Rb xenografts for FTIR analysis

Three tumor samples were randomly selected from each group. The representative mice and tumor from each group has been shown in the Fig. 1. Cryoblocks were prepared for the sectioning and cryo-sections of 7 μm were taken onto the IR-transparent calcium fluoride (CaF_2_) circular window (0.5 mm thickness by 13 mm diameter, Crystan Ltd, Dorset, UK). All tissue sections were dried in a dry cabinet before FTIR spectral data collection.

### Focal Plane Array (FPA) microspectroscopic imaging using ATR-FTIR

The FPA-FTIR study of the RB xenograft tumor samples was performed to identify the difference between the samples. FPA-FTIR micro-spectroscopic images were recorded using a Cary 620 FTIR microscope using cooled liquid-N2 environment with 128 × 128 element FPA detector objective lens, 15 × (0.62 NA) attached with FTIR spectrometer (Cary 670 FTIR spectrometer, Agilent Technologies). The FTIR Spectra were collected in a transmission mode in the spectral range between 3800-900 cm^−1^. A single FTIR image was acquired in an area of 700 × 700 cm^2^. A single FTIR spectral image contain the array of 64 × 64 spectra obtained from binning of the signal captured on detectors from each square of 4 on FPA array consist of 128 × 128 elements. A resultant single spectrum of sample collected in FTIR image acquired on ca. 10.9 × 10.9 mm^2^ revealed the molecular information about the sample functional group. From each tumor sample 5 FTIR spectral images were obtained with a resolution of 4-cm^−1^ with 128 co-added scans, Blackman-Harris 3-Term apodization, Power-Spectrum phase correction and a zero-filling factor of 2 using Resolutions Pro software package (Agilent Technologies). Before each sample measurement, background measurements were performed using a clean surface of the substrate with the same acquisition parameters^17^.

### Data processing (Statistical Analysis) by Hierarchial clustering analysis (HCA) and Principal component analysis (PCA) analysis

The protocol for the multivariate analysis of the FTIR data is shown in Flow chart (Supplementary Fig S1). The multivariate data analysis was performed using HCA CytoSpec v. 1.4.02 (Cytospec Inc., Boston, MA, USA) and PCA using Unscrambler X 10.1 software package (CAMO Sofware AS, Oslo, Norway)^18^. The quality control test was carried out to adjust the signal to noise ratio. Biobands were selected over spectral wavelengths covering 1800-930 and 3040-2810 cm^−1 ^19.

The specific spectral range was chosen to get optimal information for a range of biomolecules including lipid, protein and nucleic acids as reported previously^20^,^21^. The spectral data providing absorbance between 0.2–0.8 was used for further analysis.

The second (2^nd^) derivative was performed by a 9-point Savitzky–Golay algorithm which removed the broad baseline offset and curvature^22^. HCA analysis was carried out with Ward’s algorithm and cluster (dendrogram) imaging was carried out with second derivative spectra by randomly assigning five as the number of clusters to be generated. In addition, the final spectra were further analyzed by extended multiplicative scatter correction (EMSC). This eliminates the physical information collected from the scattering of light and removed artefacts introduced during the normalization of the path length differences. This ensured that the spectra was obtained only from the chemical information (from functional group of the biomolecules). The EMSC corrected data is a robust representation of the biomolecules^23^. After EMSC correction, the PCA was performed.

## Results

### Characterization of GNP conjugates by FTIR and Transmission electron microscopy (TEM)

The GNPs-1 and GNPs-2 conjugates were characterized by FTIR and TEM. The FTIR spectra were similar between both the conjugates and revealed six significant peaks (Supplementary Fig. S2A). The broad IR band centered at 3482 cm^−1^ is attributed to phenolic O-H stretch H-bonded. The bands at 2914 cm^−1^ and 2904 cm^−1^ are attributed to the C-H stretch of the methylene groups of 3 mercaptopropionic acid (MPA) attached to the HDM2 peptide^24^. The similarity in these peaks indicates that the MPA-functionalized HDM2 peptide is covalently bound with GNPs. The strong asymmetric band at 1645 cm^−1^signifies the carbonyl stretching of the peptide in the GNPs-1 and GNPs-2 conjugates. The series of bands between 1500 and 1200 cm^−1^ are due to the asymmetric and symmetric stretching and bending modes of aliphatic and aromatic amino acids present in the HDM2 peptide sequence (QETFSDLWKLLP). The FTIR data provides good evidence that the HDM2 peptide is conjugated with the GNPs.

The GNPs- 1 and GNPs-2 internalization was visualized using TEM after 24 h of culture in the Y79 cells to confirm the morphology, size of the particles (range of 15–20 nm) and their stability inside the cells (Supplementary Fig. S2B). TEM was carried out to characterize the conjugates inside the cells and not as conjugates themselves, as it is important to consider the stability of the GNPs inside the cellular environment^25^. This is because, the peptide coating changes the basic physiochemical interaction of GNPs by the dynamic interfaces between the nanoparticles and the biomolecules inside the cell which could affect the internalization of nanoparticles and consequently change the cellular responses. The characterization results by FTIR and TEM confirm that the HDM2 peptide is conjugated to the nanoparticles and is stable in a cellular environment (Supplementary Fig S2).

### Effect of GNP1 and GNP2 conjugates on the xenograft model

**Figure 2:**
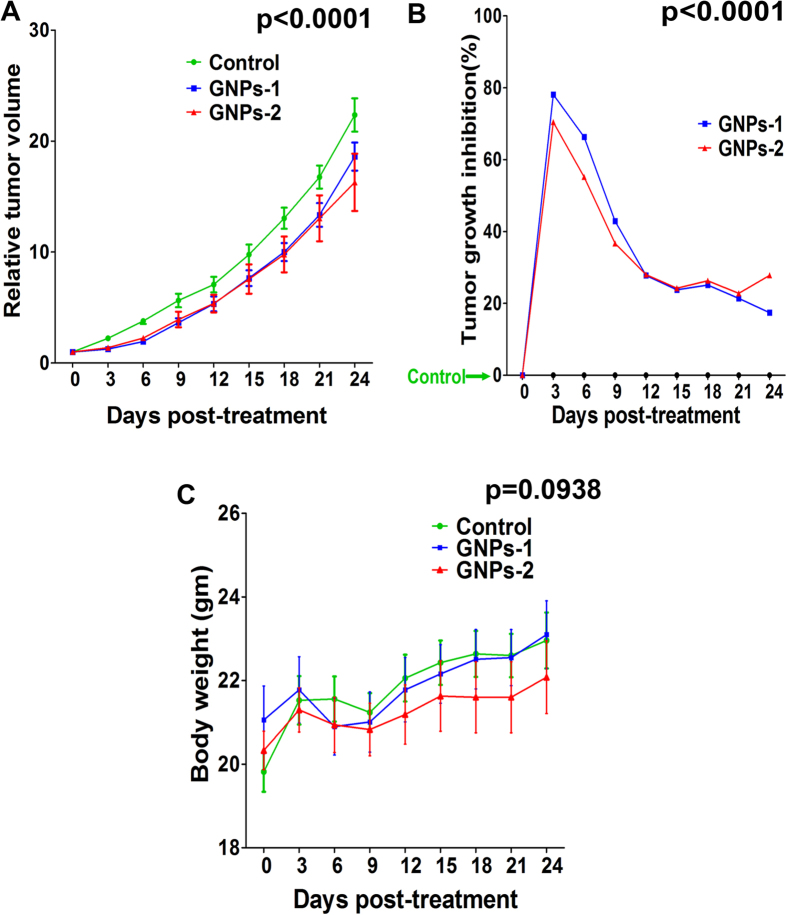
Effect of GNP conjugates on the xenograft model. The control, GNPs-1 and GNPs-2 mice were monitored every three days till 24^th^ day for tumor growth (tumor volume measured using caliper) and body weight. The relative tumor volume (RTV) and tumor growth inhibition were calculated. Mean ± SEM was calculated for the RTV data and tumor growth inhibition was presented as a percentage. Two way ANOVA was utilized for statistical analysis and p value <0.05 was considered significant. RTV (**A**) and TGI (**B**) showed statistically significant difference between the control and the treated groups (p < 0.0001). Analysis of the body weight in grams revealed that the body weight of the mice did not significantly differ between different groups throughout the treatment (p = 0.0938; Two way ANOVA) (**C**).

All the mice in the control and the treated groups survived until the end of the analysis. The mean body weight of the control, GNP1 and GNP2 treated groups at the beginning of the treatment (Day 0) were 19.82 ± 0.48, 21.06 ± 0.81 and 20.33 ± 0.46, respectively. No significant differences in the body weight were observed between the control and different treatment groups throughout the experiment (p = 0.9988; Two way ANOVA) (Fig. 2C). Analysis of the liver function (ALT, AST) and kidney function (Creatinine, BUN) tests revealed that the GNP conjugates were non- toxic to the vital organs (Supplementary Fig S3 A-D). The histopathological analysis of liver, lung, spleen, heart and kidney in all the groups appeared normal, substantiating the dose tolerance and non-toxic nature of the treatments (Supplementary Fig S4). The DLC revealed no significant changes in the percentage of eosinophils, monocytes, lymphocytes and neutrophils compared to the controls suggesting the absence of bone marrow suppression (Supplementary Fig S3E). The analysis of the relative tumor volume and tumor growth inhibition revealed that both the GNPs-1 and GNPs-2 treatment led to a statistically significant difference in the tumor reduction compared to the control (p < 0.0001; Two-way ANOVA) (Fig. 2A,B). The tumor growth inhibition at the end of 24 days were 18% and 28% in the GNPs-1 and GNPs-2 (p < 0.001) treated groups, respectively.

#### **Hierarchical clustering analysis of the FTIR results**

**Figure 3:**
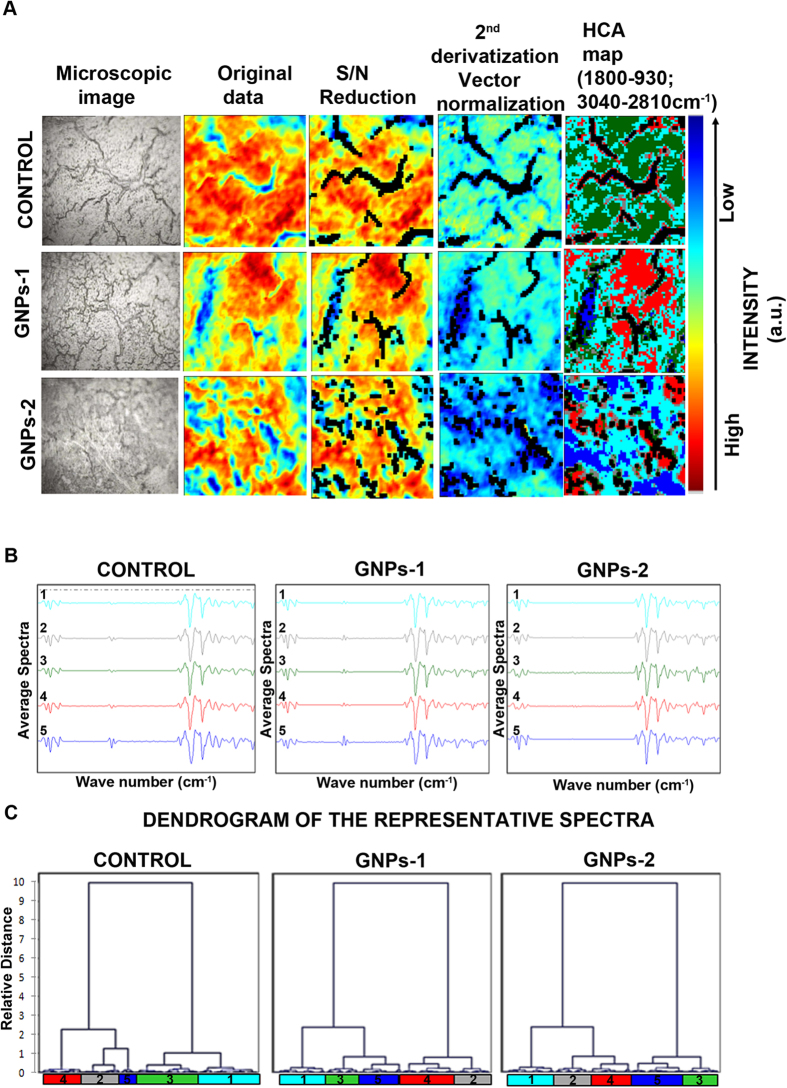
Hierarchial clustering analysis (HCA) of the FTIR data. FPA-FTIR microspectroscopic images of the tumors obtained from each group (Control, GNPs-1 and GNPs-2) were subjected to quality test and signal to noise (S/N) reduction followed by 2^nd^ derivatization and vector normalization. HCA map was generated from the pre-processed 2^nd^derivative data (**A**). HCA dendrogram was obtained by Ward’s algorithm and squared Euclidean distance measure criterion, using the entire dataset that included the five clusters in each samples. The average spectrum (**B**) and the dendrogram (**C**) for the Control, GNPs-1 and GNPs-2 are provided. The Y axis denotes the relative distance and X axis denotes the 5 different clusters corresponding to the spectra which are hypothesized to arise from five different kinds of cells in the tumor tissue.

The cluster analysis was performed on pre-processed “2^nd^ derivative” data of the area-normalized spectra in the region 3040-930 cm^−1^. The spectral range of 1800-930 and 3040-2810 cm^−1^ was obtained after vector normalization (Fig. 3A). Discrimination between the three samples in a hierarchical tree was made with multi-dimensional HCA using Ward’s algorithm, which is the most appropriate method for quantitative variables and clustering into group^26^. The HCA analysis allows for the visualization of the group and sub-group arrangement of the spectra. The HCA revealed the intragroup similarity within untreated control GNPs-1 and GNPs-2 groups and generated-5 random clusters in each group (Fig. 3C). The difference/similarity is set arbitrarily to 5 different spectra which are hypothesized to arise from five different kinds of cells of the cancer tissue due to its heterogeneous nature (Fig. 3B). These spectra were then kept constant between the samples analyzed.

The difference in the spectral range was established by considering similar areas of biological functional groups in all the samples with the highest possible difference between same tumor samples. The dendrograms clearly showed distinct clusters in the control, GNPs-1 and GNPs-2 treated RB xenograft tumor sample (Fig. 3C). The results suggested that the biochemical composition of RB xenograft tumor sample was differently influenced by the treatment of the two GNP-1 and GNPs-2 compared to the control group. While, the HCA dendrogram indicates differences in the treated (GNPs-1 and GNPs-2) and untreated groups, there are important questions that are left unanswered for example What are the variations in the biochemical functional group between the controls and treated that brings about the difference in the HCA analysis? Also how do vibrations of different functional groups in biomolecules vary in terms of their intensity and shift? These questions need to be considered for the sensitivity and specify the measurement of the FTIR data. Therefore, PCA was used further to get an insight into the above mentioned questions.

#### **Principle Component Analysis (PCA).**

PCA was performed to extract the most significant difference in the spectra generated between the three groups (control, GNPs-1 and GNPs-2). PCA was performed on the absorbance spectra of the data sets obtained from all the three groups before and after 2^nd^ derivatization.

#### **PCA analysis in bio-band range before 2^nd^ derivatization.**

**Figure 4:**
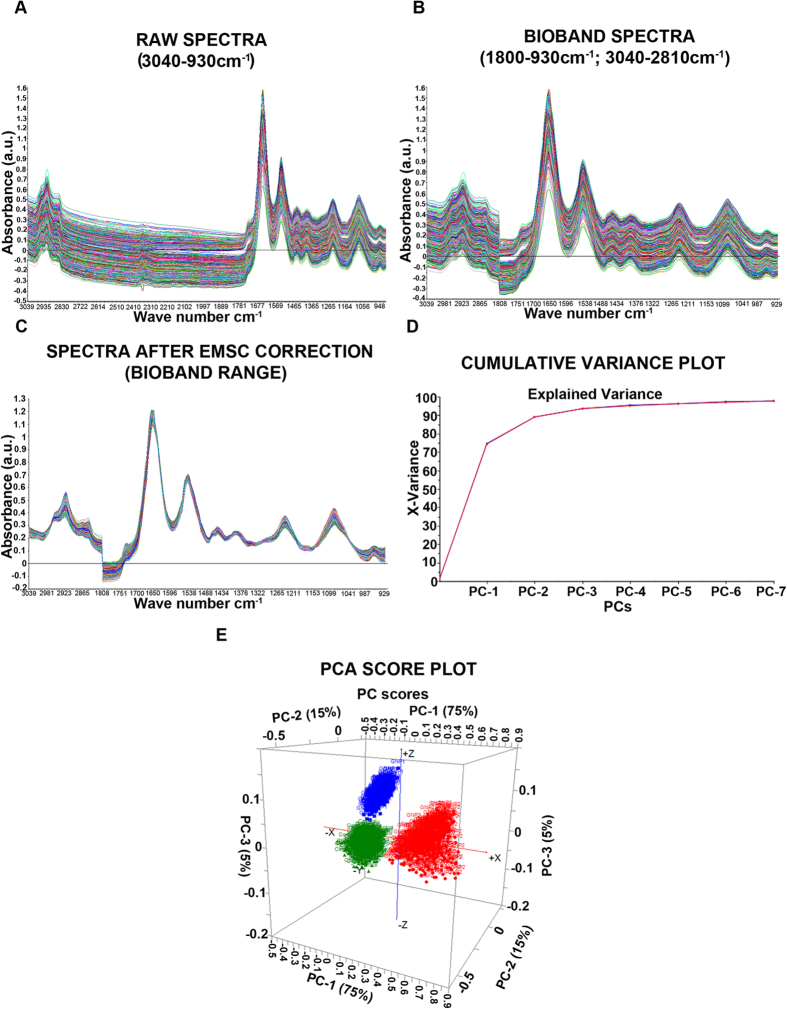
Principal component analysis (PCA) on the raw spectral data before 2^nd^ derivatization. The raw spectral data (wavelength range 3040-930 cm^−1^) (**A**) was pre-processed to obtain the “bioband” spectra (wavelength: 1800-930 cm^−1^; 3040-2810 cm^−1^) corresponding to the major biomolecules such as proteins, lipids and nucleic acids (**B**). The spectra corresponding to bioband range was further analyzed by extended multiplicative scatter correction (EMSC) to obtain only the spectra from chemical information (**C**). PCA analysis of the EMSC corrected spectra revealed maximum difference in the PCs between the Control, GNPs-1 and GNPs-2 as revealed by the Cumulative variance plot (**D**) and PCA score plot (**E**).

A total of 1493 raw spectra were obtained between wavelengths (3040-930 cm^−1^) from all three groups (Fig. 4A). This included 543 from vehicle control, 486 from GNPs-1 and 464 from GNPs-2. This data was further analyzed in the bioband range (1800-930 and 3040-2810 cm^−1^) (Fig. 4B) followed by the EMSC correction (Fig. 4C) and PCA analysis on the raw data (Fig. 4D,E). PCA was performed on the spectral data of “BioBands” through cross validation at 7 PCs. The interval of the wavelength was kept at 1–60 and 320–548 in the ranges of wave numbers: 3040-2810 cm^−1^ and 1800-930 cm^−1^, respectively. The actual bioband wave number was 3039.234 - 2811.677 and 1808.883 - 929.51 cm^−1^. The specific range was selected since, it is indicated in the identification of lipids, nucleic acid and protein molecules present in the mammalian cell^27^,^28^.PCA was applied to all the data and provided 3 levels with 95.22% of variance, best observed in three-dimensional space (PC1 versus PC2 versus PC3) (Fig. 4D,E). The PC score plot of unpriced original data showed clear scattering at different PC levels for all the three groups studied.

Since, there was a higher number of spectral band overlap in the unprocessed spectrum, it was necessary to process the data and use the second derivative to get the distinct individual bands from the original data set. Hence, for the optimal analysis, the raw data was subjected to 2^nd^ derivatization.

#### **PCA analysis in bio-band range using data obtained after 2^nd^ derivatization.**

**Figure 5:**
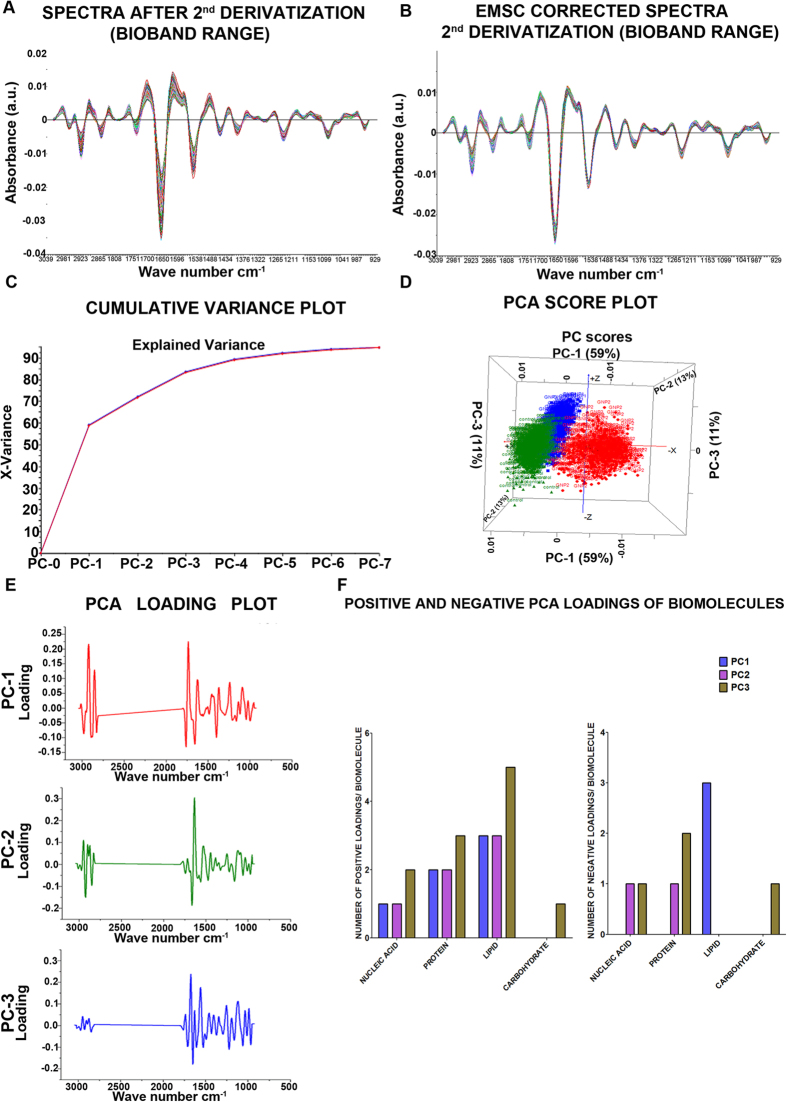
Principal component analysis (PCA) on the 2^nd^ derivative spectra. The 2^nd^ derivative spectra (**A**) was analyzed by EMSC to obtain the spectra from chemical information (**B**). The cumulative variance plot (**C**) and the PCA score plot (**D**) revealed maximum variance between the three groups control (PC2), GNPs-1(PC3) and GNPs-2 (PC1). The PCA loading plot showed maximum difference in the bioband range between the three groups (**E**). The graph summarizing the biomolecules in both positive and loading with respect to all the three PC level is provided (**F**).

In the pre-processed data before 2^nd^ derivative, an overlap of the vibrations of cellular biomolecules is expected and the result derived may reflect the average biochemical information. Second (2^nd^) derivatization was performed with 13-point smoothing and 3 polynomial order (Fig. 5A) to get the sub-band which may have been contributed as an average band in the raw data set. The EMSC correction (Fig. 5B) was applied to the 2^nd^ derivatization data before PCA analysis. The cumulative variance and score plots (Fig. 5C,D) aid in the visualization of the data in PCA, whereas; the loading plot (Fig. 5E) is an indicator of the biochemical functional group obtained at PC level^29^. The PC loading reveals the differences derived from unique signatures pertaining to the vibration of various biomolecules which are a source of variation between samples (Fig. 5E). PCA analysis revealed a clear discrimination between the three groups, which is a function of the PC variation in the samples. It was observed that at PC-1 (GNPs-2) and PC-3 (GNPs-1) more loading was observed with respect to the control (Fig. 5E). These differences are probably due to the differences in the treatment of GNPs-1 and GNPs-2. We attempted to interpret each PC loading in terms of the difference in positivity and negativity loading of the functional group (Table 1). The difference in the PC corresponding to GNPs-1 (PC3) and GNPs-2 (PC1) could be attributed to the different chemical used for synthesis of GNPs (Fig. 5E). The extent of PC loading (both positive and negative) was highest in the PC3 corresponding to GNPs-1 and lowest in PC1 corresponding to GNPs-2 in the bioband range 1800-930 cm^−1^ (actual range: 1808.883-929.511 cm^−1^)whereas the extent of PC loading was more in PC2 corresponding to control compared to GNPs-1 and GNPs-2 in the bioband range 3040-2810 cm^−1^ (actual range: 3039.234-2811.677 cm^−1^) (Fig. 5E).

The PC-3 corresponding to GNPs-1 showed the highest number of loading both positive and negative compared to GNPs-2 and control (Table 1). The GNPs-1 is reduced with the *Vitis vinifera* which consists of polyphenols and hence resulted in higher biochemical changes in a wide range of the macromolecules such as lipids, protein, and nucleic acid. Alternatively, GNPs-2 synthesized with the sodium citrate showed interaction mostly with the lipid molecules and could justify the reduction in loading as observed in PC-1 (Fig. 5E).

The difference in the loading observed between the groups could be correlated to the biological responses such as difference in tumour growth reduction (as explained above, Fig. 2); and the mechanism underlying the response to treatment. The real-time PCR and apoptotic array analysis were carried out to find the mechanism behind the responses to the different treatments. The real time PCR analysis of the control, GNPs-1 and GNPs-2 groups revealed that the transcript levels of p53 was significantly upregulated in the GNPs-1 while significantly down-regulated in the GNPs-2 group compared to controls (Supplementary Fig S5A). In addition, the transcript levels of HDM2 were found to be significantly downregulated in both the treated groups compared to control (Supplementary Fig S5B). This suggested that the GNPs-1 and GNPs-2 were going through p53 dependent and p53 independent pathways, respectively. The apoptotic array analysis on the three groups showed differences in 17 out of 35 proteins analyzed (Supplementary Fig S5C). The targets of p53 such as FAS^30^,^31^, FADD^31^ and TRAILR2^32^ were significantly upregulated in GNPs-1 group with a consequent increase in the procaspase 3 and cleaved caspase 3 suggesting p53 dependent apoptotic pathway. The phosphorylated p53 levels were not significantly upregulated in these groups. A recent study suggests that phosphorylation of p53 is dispensable for both transcriptional activation and apoptosis^33^

On the other hand in the GNPs-2, the p53 dependent proteins were not significantly different. In addition, Bcl2, a pro-apoptotic protein level was significantly down regulated in the GNPs-2 treated group without an increase in the caspases. Recent studies on the inhibition of Bcl2 shows that the suppression of Bcl2 in the absence of caspase dependent apoptosis leads to autophagic cell death in leukemia and breast cancer^34^,^35^. In addition, HIF 1 alpha was also found to be down regulated in the GNP2 group. It has been shown previously that inhibition of HDM2 by nutlin3 an antagonist leads to downregulation of HIF 1 alpha even in p53 null cells which eventually leads to VEGF suppression^36^. Suppression of VEGF in retinoblastoma has earlier been shown to inhibit cell tumorigenesis^37^.

However, in both the treatments, Hsp60 and survivin levels were found to be significantly upregulated compared to control, suggesting that the HDM2 peptide may not be effective in controlling this pathway that may have reduced the efficacy of the current treatment (Supplementary Fig S5C). Hence, the differences in the PC loading that were observed with the FTIR data could be attributed to the mechanism leading to the therapeutic responses.

After the overall analysis of the PC loadings, the results of the different bands at different PCs level were analyzed (Table 1). To set the difference in PC loading the most significant positive and negative loadings (both the intensity and shift in the spectral peak) were considered in the treated (GNPs-1 and GNPs-2) and untreated (Control) groups. A reduction in the PC loadings between the wavelengths 3400 to 2800 was observed in GNP-1 compared to the control and GNP- 2 groups. This specific range of wavelength (3700 to 2800) representative of the lipids is indicated in malignancy^19^. Hence, the observation of reduction in this PC loading indirectly might indicate the response to treatment with regard to the GNP-1, which might imply the use of this spectral signature as a potential marker in the specific therapeutic scenario^38^. However, the therapeutic response could also be appreciated in GNPs-2 which did not show significant PC loading difference in the above-mentioned wavelength compared to the control. There was a shift in the wavenumber corresponding to CH2 anti-symmetric and CH2 symmetric stretching of lipids in GNP-2 compared to control (Table 1). While the CH2 anti- symmetric stretching was observed at wavenumber 2937 cm^−1^ in case of the control (PC2); a shift towards a lower wavelength 2929 cm^−1^ and increase in intensity was observed in case of GNPs-2 (PC1); it is hypothesized that the spectral shift and change in the intensity may reflect the response to the specific treatment with GNPs-2.

Similarly, signal in the wavenumber range 1700 cm^−1^ to 1600 cm^−1^; corresponding primarily to the proteins amide I functional group were present in all the three groups with a difference in the intensity in the order of PC2 > PC3 > PC1. While the amide I functional group was due to the C = O stretching of the peptide bond with a secondary conformation of β-sheet in the control (PC2) and GNPs-2 (PC1); the same was enriched as β-turn conformation in the GNPs-1 (PC3) group. The analysis of wavelength suggested that there was a significant shift in case of GNPs-1 (PC3) (1675) compared to the control (PC2) (1625) and GNPs-2 (PC1) (1622). In addition, in the case of the control (PC2), the amide I with the secondary structure of β-turn conformation was increased in the negative loading. In addition, the functional group corresponding to amide III β-sheet was present in the positive loadings of the treated groups GNPs-1 (PC3) and GNPs-2 (PC1) and not in the control (PC2). However, there was a wavelength shift which differentiated the different treatments (Table 1). It is hypothesized; that the spectral signature corresponding to the amide III might serve as a diagnostic marker for monitoring the therapeutic response irrespective of the GNPs.

With respect to the phosphate group containing biomolecules such as phospholipids, nucleic acids and phosphorylated polysaccharides a significant difference in the positive loading was observed in the wavenumber range 1100-800 cm^−1^ between the treated group (GNPs-1 (PC3) and GNPs-2 (PC1)) compared to the control group (PC2).The decrease in the phosphate group containing molecules are reported in cancer tissue as a diagnostic marker^39^,^40^. Hence, their enrichment could be attributed to the response to the therapy. Together, the results suggest that the amide III β sheet and the phosphate group containing biomolecules might serve the purpose of monitoring the response to functionalized GNP based therapies.

## Discussion

FTIR spectroscopy is currently being employed as a tool to derive biochemical signatures for a wide range of cells and tissues^41^. FTIR has been used to distinguish the normal and cancer cells due to the presence or absence of macromolecules such as nucleic acids, proteins, lipids and carbohydrates and differences in their configurations^42^,^43^,^44^. FTIR has been useful in identifying the differences in complex cancer tissue as variations in the spectral profiles contributed by specific vibrational peak shifts, band shapes and intensity. The spectroscopic methodology could be a new diagnostic tool for the analysis of the tumor sample due to sensitivity and specificity without interference of chemicals used in the processing of the tissue for the analysis unlike IHC where interference can be a problem^45^. In this study, we explored the possibility of utilizing FTIR in place of the conventional techniques for investigating the therapeutic response and mechanisms involved in nanoparticle mediated targeted therapy (GNPs-HDM2) in a RB xenograft mouse model. Comparison of the FTIR spectra for PC-1 to PC-3 clearly revealed the difference induced by GNPs-2 and GNPs-1. From these results, we can hypothesize that a targeted moiety can interact with the different biomolecules during the treatment. As we have noticed here, although a peptide (HDM2) was used for the targeting the oncoprotein molecule, due to difference in the carrier molecules (GNPs) the interactions were not limited to the protein and a significant difference was observed in interactions with various biomolecules as revealed by the PCs loading (Fig. 5F) compared to the control samples. As discussed, GNPs-2 showed loadings predominantly corresponding to lipids, whereas GNPs-1 showed significant loadings corresponding to proteins with little or no lipids. The difference in the specific band loading is a means to identify the difference of the biomolecules. In a similar study FTIR analysis to differentiate between normal and a mutated genome was used to screen the mutagenic potential effect of the drug^46^. The application of FTIR in combination with specific biological assays can be utilized for assigning the differential band position and intensity to specific molecular events between compared samples^47^.

In addition, most of the research studies widely use molecular biological techniques such as gene expression and protein expression studies to analyze the mechanism of action of drugs, peptides or siRNA^13^. For instance, the validation of peptide or siRNA based experiments is limited to protein expression, while the differences in the other biomolecules are ignored. Since, biological systems are complex, modulation of one specific group of biomolecule (e.g. protein) can cause a cascade of changes that does not limit itself to target groups such as protein. Hence a method that would provide a holistic view of interactions between different biomolecules are vital^48^. Application of FTIR fulfills the above mentioned criteria. Our results reiterate the need for a method like FTIR for understanding the complete array of biomolecular interactions. Additionally, the combination of FTIR with multivariate analysis could provide a non-invasive and reproducible method for assessing the differences between untreated/treated, cancerous/non-cancerous samples. The transformation of the tissue states from normal to cancer of treated to untreated is attributed to biochemical changes within the tissue. Since the tumor is an abnormal stage of the cell associated with chemical changes at the molecular level, the tumor cells can be monitored in comparison with the normal cells based on their chemical composition, thereby giving vital information with regard to early diagnosis of the tumor stages, benign or malignant. As an extension, the technique has the ability to provide biologically relevant data which could be clinically significant in diseases like cancer which are highly heterogeneous; thereby aiding in rationalizing the treatment plan and drug designing. Furthermore, FTIR can be applied *in vivo*/*in situ* for monitoring the progression of cancer and its response to therapy without the requirement for obtaining tissue biopsy for histochemical analysis which makes it the preferred method due to its non-invasiveness and rapidity^49^,^50^.

## Conclusion

The advent of FTIR with multivariate analysis has revolutionized the field of cancer. Conservatively, cancer biomarkers or targets are inferred as single or multiple genes, proteins or other biomolecules which are identified by specific molecular or integrated “omics” approach. FTIR offers unique advantages as it reflects the overall vibrations of the cellular components and their interactions within the samples as spectra in addition to being non-invasive and label-free, unlike conventional molecular methods. FTIR has been utilized in the field of oncology to distinguish normal and cancerous tissue, and for grading cancers which allow for treatment decisions. In this study, we utilized RB xenograft model and nanoparticle based therapy to establish the utility of FTIR. The technique was capable of differentiating the mechanistic and therapeutic response in the treated and untreated groups as variations in their spectral signatures which corresponds to the differences in their biomolecular fingerprint. In total, the current finding expand the potential use of the FTIR- technique in the field of cancer therapeutics primarily where the focus is on interactions between a drug (here nanoparticle) and the target molecules. Future experiments should include longitudinal studies involving temporal analysis of tumor growth as regression and/or relapse in the animal models of cancer can aid in the monitoring of therapeutic response using FTIR.

## Additional Information

**How to cite this article**: Kalmodia, S. *et al.* Attenuated Total Reflectance Fourier Transform Infrared Spectroscopy: An analytical technique to understand therapeutic responses at the molecular level. *Sci. Rep.* **5**, 16649; doi: 10.1038/srep16649 (2015).

## Supplementary Material

Supplementary Information

## Figures and Tables

**Table 1:** Major spectral peaks identified in different treatment groups and their functional group assignments^17^,^49^,^50^.

Level of PC	Loadings Wavelength (cm^−1^)
PC-1 (+)	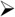 1092 -PO_2_^−^ group of DNA/RNA and phospholipids backbone
	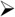 1242- β-sheet of amide III bond
	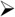 1622- Antiparallel β-sheet, _V_(C = O) of carboxylate and _V_(C = C) of aromatic compounds of amide I bond
	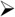 1738- _V_(C = O) bond of esters group from fatty acids and lipid triglycerides
	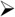 2847- _V_s(C–H) bond of methylene (–CH2) of lipids
	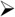 2929- _V_as(C–H) bond of methylene (–CH2) of lipids
PC-1 (−)	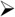 1758- _V_(C = O) bond of esters group from fatty acids and lipid triglycerides
	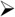 1649- _V_(C = C) of di substituted cis-olefins and α-helix of amide I
	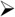 1388- ^δ^s(CH3) and ds(CH2) of lipids and proteins
PC-2 (+)	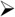 1377- ^δ^s(CH3) from cholesterol and fatty acid radicals
	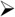 1441- _V_(C–N) of the pyridine ring
	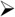 1526- Parallel mode of the α-helix of amide II
	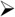 1625- Antiparallel b-sheet of amide I, _V_(C = O) of carboxylate and _V_(C = C) of aromatic compounds
	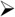 2851- _V_s(C–H) from methylene (–CH2) groups of lipids
	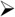 2937- _V_as(C–H) from methylene (–CH2) groups of lipids
PC-2 (−)	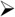 1675- β-turn of amide I
	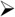 1150- _V_as(CO–O–C) group of glycogen and nucleic acids (DNA and RNA)
PC-3 (+)	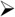 982- _γ_( = CH) of trans isomers and conjugated trans, vibration involving OH group of ribose rings in RNA
	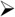 1029- _V_(C–C) coupled with ^δ^(CH2) of ^α^CH2 in –CH2OH groups of polysaccharides
	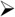 1109- _V_s(C–O) at the 2′-OH group of ribose rings in RNA
	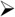 1249- β-sheet of amide III
	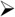 1342- _γ_wag(CH2) of ^α^CH2 groups in polyethylene chains
	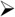 1416-^δ^rock(CH2) of di-substituted cis-olefins
	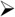 1468-^δ^scissor(CH2) from methylene (–CH2) groups in acyl chains of lipid bilayers in orthorhombic packing
	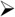 1555- α-helix and antiparallel β-sheet of amide II
	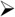 1675- β –turn of amide I
	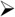 2888- _V_s (C–H) from methyl (–CH3) groups of lipids
	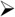 2948- _V_as(C–H) from methylene (–CH2) groups of lipids
PC-3 (−)	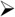 1649- _V_(C = C) of di-substituted cis-olefins and α-helix of amide I
	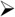 1529- Parallel mode of the α-helix in amide II
	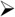 1056- _V_s (R–O–P–O–R) from ring vibrations of carbohydrates
	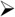 1229- -(PO_2_^−^) PO_2_^−^ group of DNA/RNA and phospholipids backbone

Abbreviations: _V_-Stretching Vibration; _Vs_–Symmetric Stretching Vibration; _Vas-_Asymmetric Stretching Vibration, δ- in plane bending vibration.
