# Supplementary material for: Attenuated Total Reflectance Fourier Transform Infrared Spectroscopy: An analytical technique to understand therapeutic responses at the molecular level
Source: Sci Rep. 2015 Nov 16;5:16649. doi: 10.1038/srep16649 (PMC4645174; doi:10.1038/srep16649)
Supplement: Supplementary Information [file srep16649-s1.pdf]

**Attenuated Total Reflectance Fourier Transform Infrared Spectroscopy: An analytical technique to understand therapeutic responses at the molecular level**

Sushma Kalmodia[1,2], Sowmya Parameswaran[3], Wenrong Yang[2], Colin J. Barrow[2],and Subramanian Krishnakumar[1*]

[1]Department of Nano biotechnology, Vision Research Foundation, Sankara Nethralaya, 18, College Road, Nungambakkam, Chennai – 600 006, India.

[2]Centre for Chemistry and Biotechnology, Deakin University, Geelong campus, VIC 3216, Australia

[3]Radheshyam Kanoi Stem Cell laboratory, Vision Research Foundation, Sankara Nethralaya, 18, College Road, Nungambakkam, Chennai – 600 006, India.

[*]Corresponding Authors: Dr. S. Krishnakumar,

Dept of Nano biotechnology, Vision Research Foundation, Sankara Nethralaya, 18, College Road, Nungambakkam, Chennai – 600 006, India. Ph: +91-44-28271616; Extn: 1358, Fax: +91-44-28254180

E-mail: drkrishnakumar_2000@yahoo.com; drkk@snmail.org

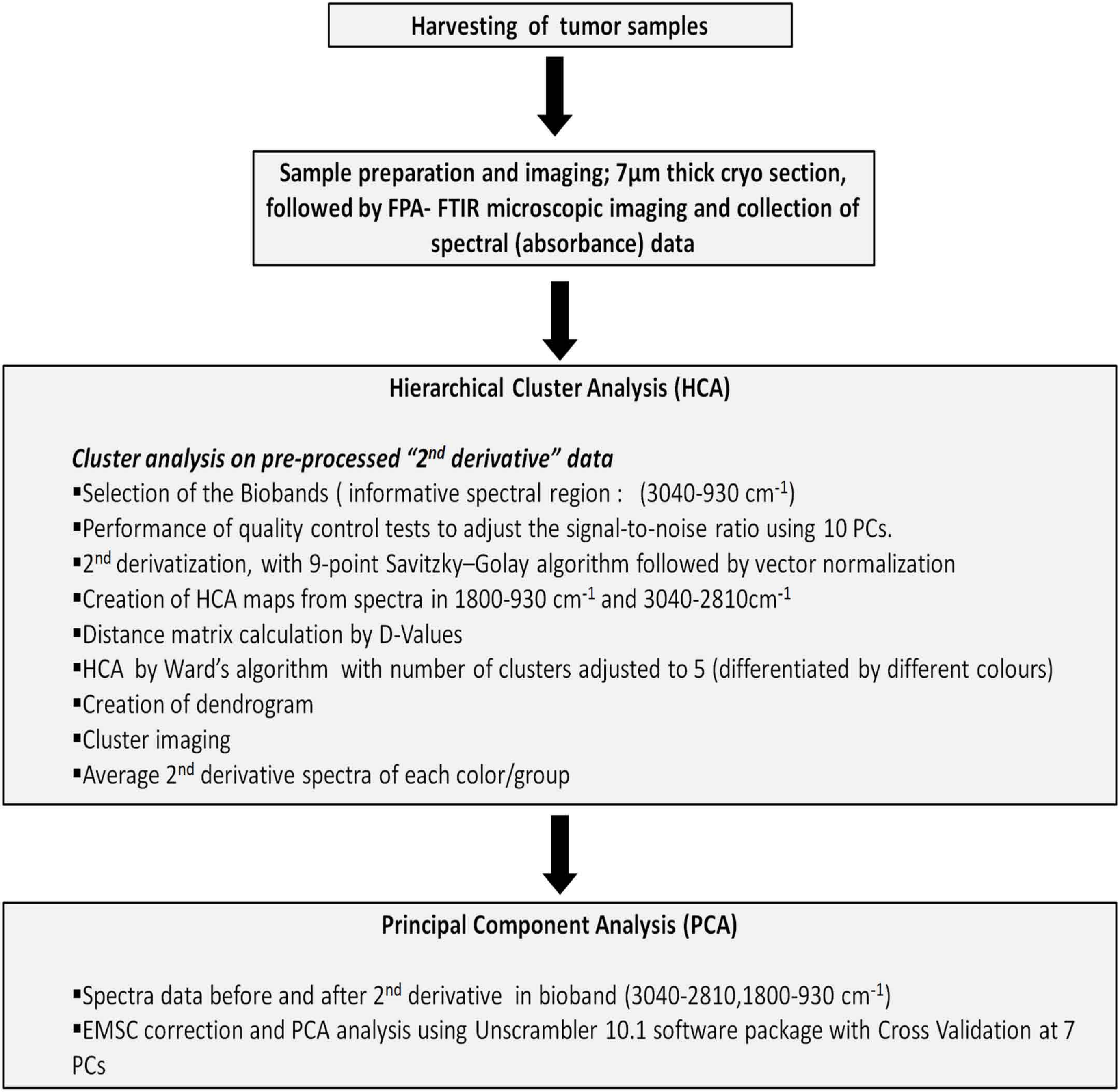
Harvesting of tumor samples
Sample preparation and imaging; 7µm thick cryo section, followed by FPA- FTIR microscopic imaging and collection of spectral (absorbance) data
Hierarchical Cluster Analysis (HCA)
Cluster analysis on pre-processed "2nd derivative" data
▪Selection of the Biobands ( informative spectral region : (3040-930 cm-1)
▪Performance of quality control tests to adjust the signal-to-noise ratio using 10 PCs.
▪2nd derivatization, with 9-point Savitzky–Golay algorithm followed by vector normalization
▪Creation of HCA maps from spectra in 1800-930 cm-1 and 3040-2810cm-1
▪Distance matrix calculation by D-Values
▪HCA by Ward's algorithm with number of clusters adjusted to 5 (differentiated by different colours)
▪Creation of dendrogram
▪Cluster imaging
▪Average 2nd derivative spectra of each color/group
Principal Component Analysis (PCA)
▪Spectra data before and after 2nd derivative in bioband (3040-2810,1800-930 cm-1)
▪EMSC correction and PCA analysis using Unscrambler 10.1 software package with Cross Validation at 7 PCs

**Figure S1**

# FTIR OF GNP CONJUGATES

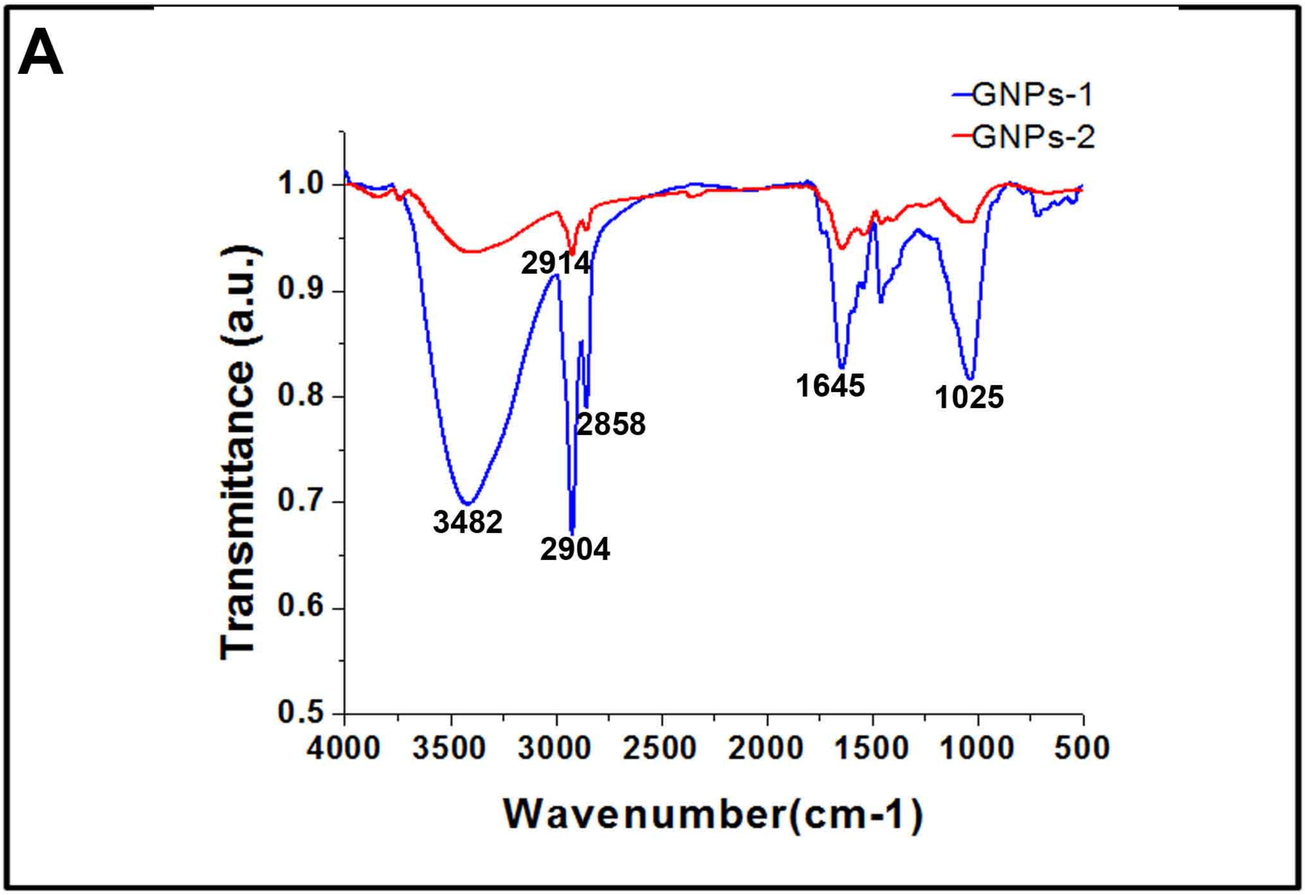


# ELECTRONMICROGRAPH OF GNP CONJUGATES

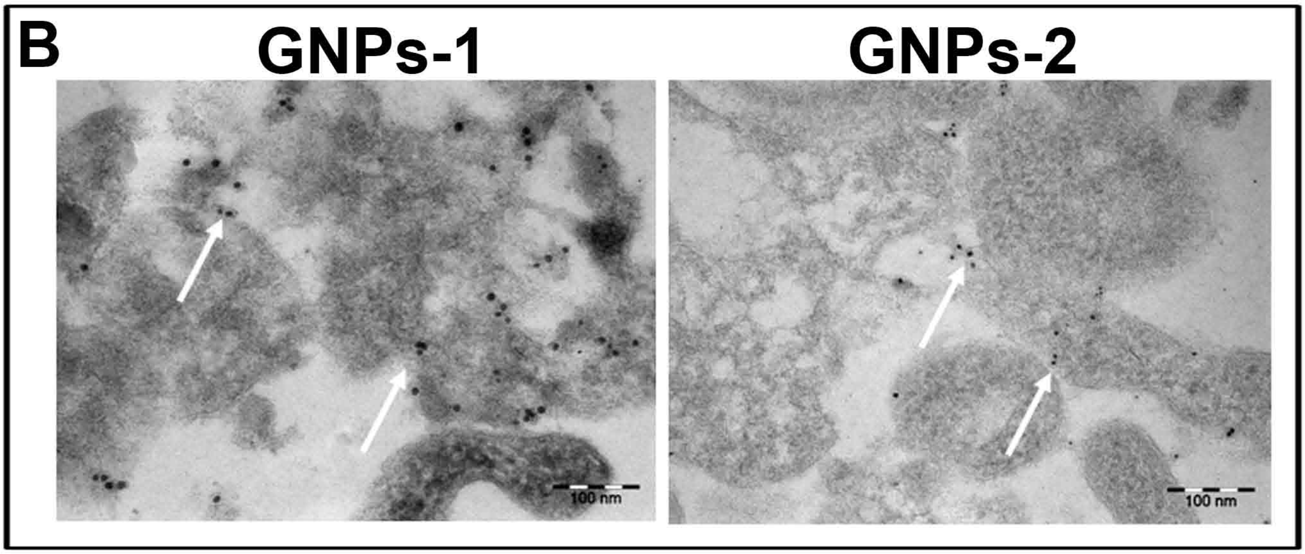


Figure S2

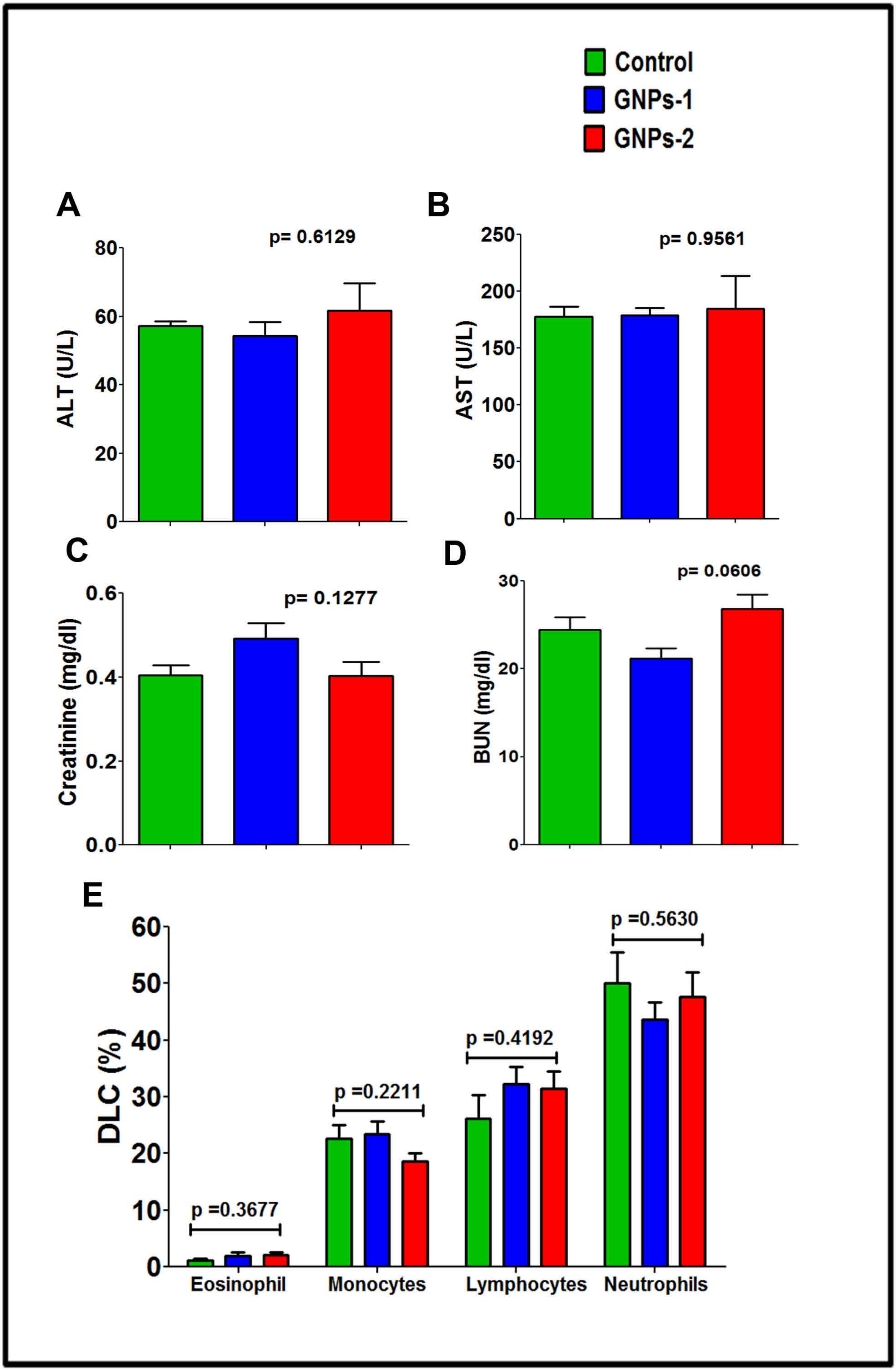

Control
GNPs-1
GNPs-2
A
p= 0.6129
ALT (U/L)
80
60
40
20
0
B
p= 0.9561
AST (U/L)
250
200
150
100
50
0
C
p= 0.1277
Creatinine (mg/dl)
0.6
0.4
0.2
0.0
D
p= 0.0606
BUN (mg/dl)
30
20
10
0
E
DLC (%)
60
50
40
30
20
10
0
p =0.3677
p =0.2211
p =0.4192
p =0.5630
Eosinophil
Monocytes
Lymphocytes
Neutrophils


# Figure S3

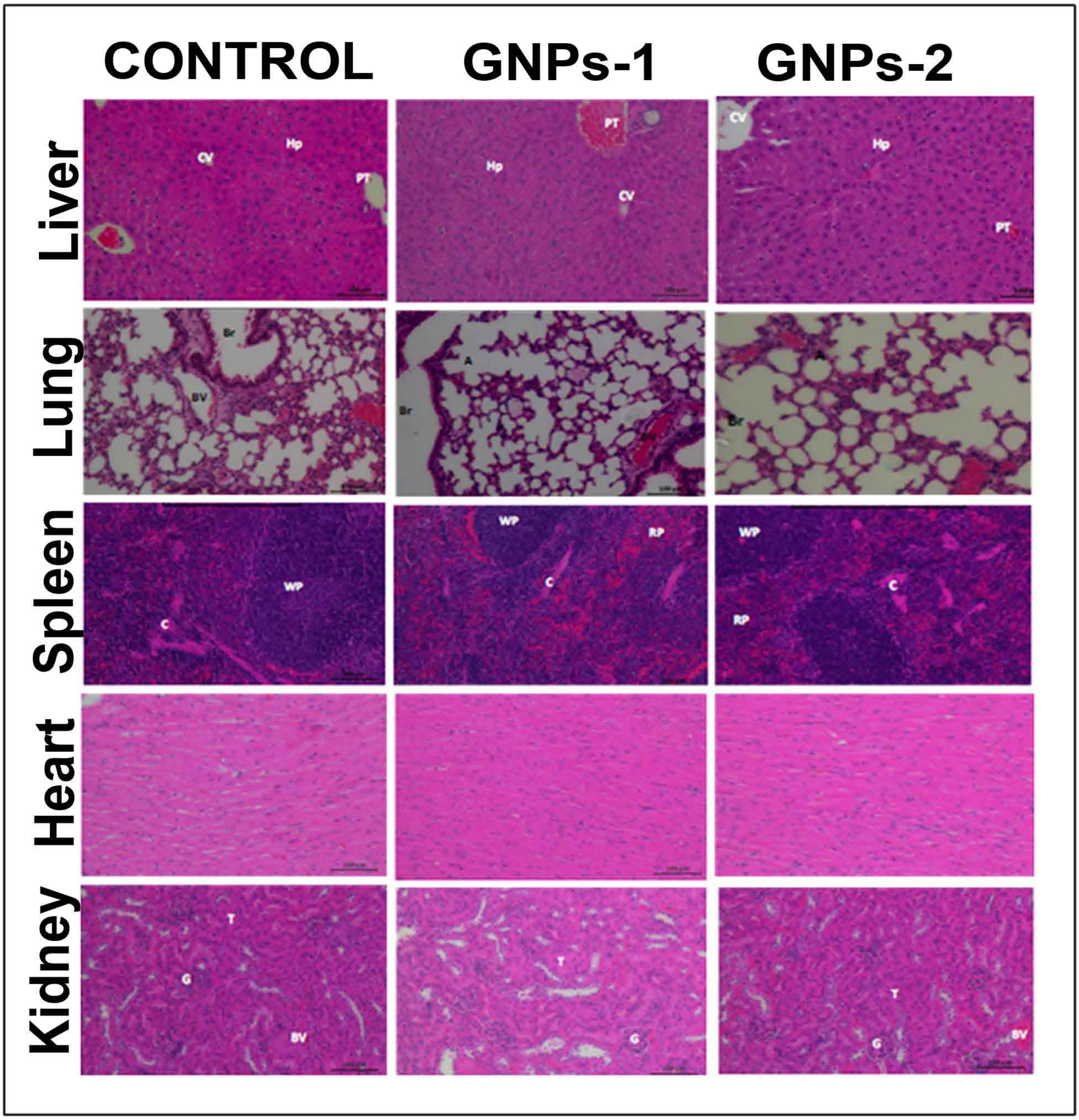
CONTROL
GNPs-1
GNPs-2
Liver
Lung
Spleen
Heart
Kidney
CV
Hp
PT
Br
BV
A
WP
RP
C
T
G

**Figure S4**

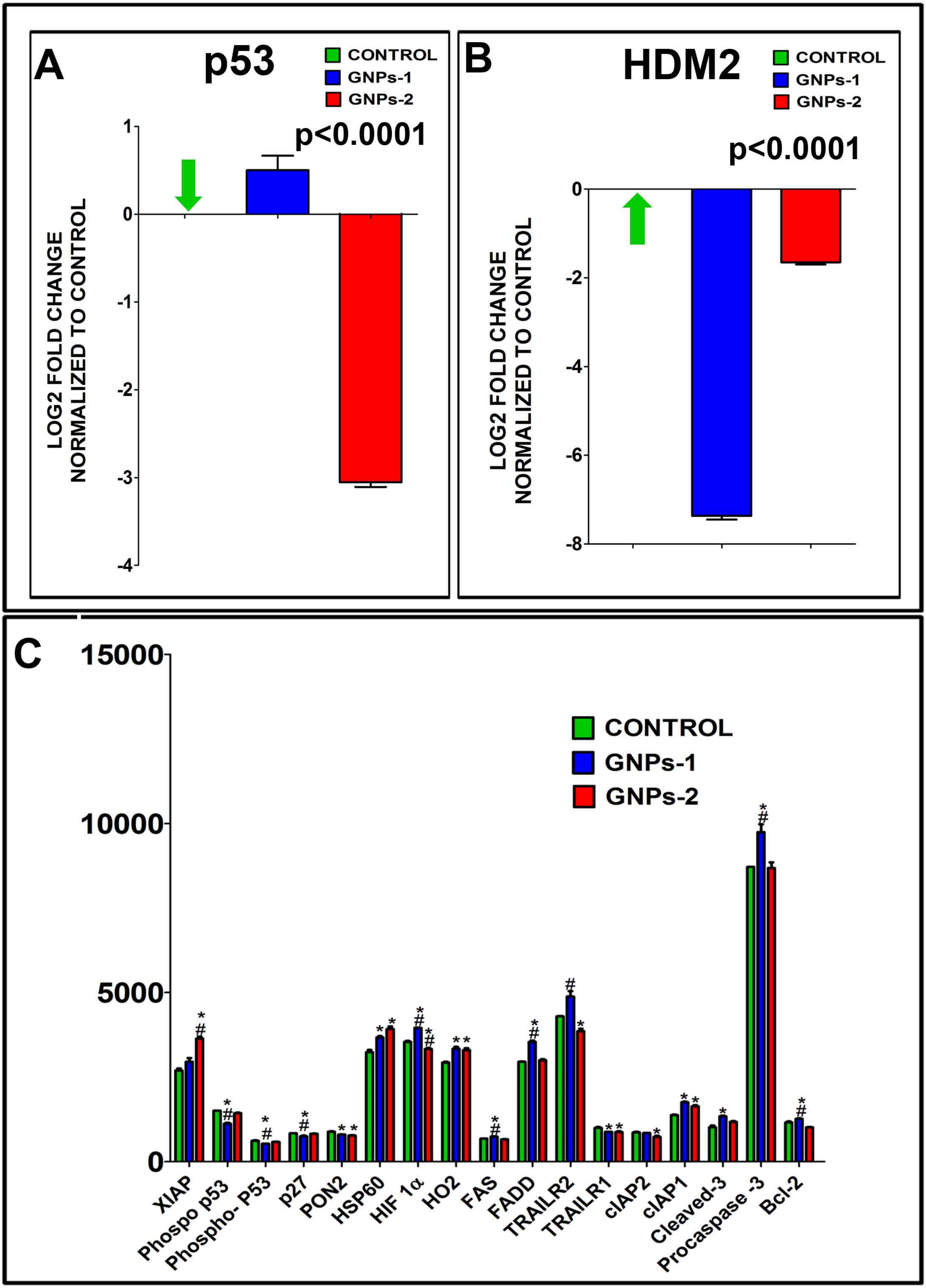

A
p53
CONTROL
GNPs-1
GNPs-2
p<0.0001
LOG2 FOLD CHANGE
NORMALIZED TO CONTROL
1
0
-1
-2
-3
-4
B
HDM2
CONTROL
GNPs-1
GNPs-2
p<0.0001
LOG2 FOLD CHANGE
NORMALIZED TO CONTROL
0
-2
-4
-6
-8
C
15000
10000
5000
0
CONTROL
GNPs-1
GNPs-2
XIAP
Phospo p53
Phospho- P53
p27
PON2
HSP60
HIF 1α
HO2
FAS
FADD
TRAILR2
TRAILR1
cIAP2
cIAP1
Cleaved-3
Procaspase -3
Bcl-2


# Figure S5

**Supplementary methods:**

**Fourier Transform Infrared Spectrum (FTIR):**

The FTIR was performed in the range of 500-4000 cm-1 using the transmittance mode by potassium bromide (KBr) pellet method in FTIR instrument (Vortex 70, BRUKER).

**Transmission electron microscopy (TEM):**

The internalization study was performed using Y79 cells *in vitro*. The exponentially grown Y79 cells (Section 3.4.1), of density $1x10^6$, were cultured in 6 well plate. After 12 h of culture, cells were treated and incubated with 50µM/well of GNP conjugates for 24h. After incubation, the cells were washed thrice with PBS and the resultant cell pellets were fixed in 2.5% glutaraldehyde prepared using 0.1M sodium cacodylate buffer (pH 7.2-7.4) and samples were processed for further analysis.

The fixed cells were washed with 0.1M sodium cacodylate buffer thrice and fixed for 2h at $8^0$C using 0.1% Osmium tetroxide (OsO4) prepared in 0.1M sodium cacodylate buffer. The excess fixative was removed and dehydrated with a series of acetone from 30%, 50%, 70%, 80%, 90% (v/v) for10 minutes in each concentration followed by 100% acetone wash twice for 10 minutes. This was followed by propylene oxide treatment twice. The cells were then infiltrated with the resin mixture and acetone, where the resin mixture consisted of Epon 812 resin, DDSA (Dodecenyl Succinic Anhydride) and NMA (Nadic® Methyl anhydride) starting with 25%, 50% and 75% for 2 hours in each concentration and then finally with 100% resin twice overnight. The final infiltration was carried out in the resin mixture with added catalyst (DMP 30) in “easy molds” at 60°C for 48 hours followed by sectioning. The resin blocks were removed from the mold, trimmed and sectioned using Leica Ultra cut R ultramicrotome with diamond or glass knives. The semi-thin sections were cut and stained with toluidine blue and screened using the

light microscope to check for area of interest followed by ultrathin sections. The sections were collected on a copper grid with 300 mess size and stained with saturated solutions of uranyl acetate followed by lead citrate. The samples were air dried and was screened in JEOL JEM 1400 transmission electron microscope. TEM was operated at an accelerating voltage of 80 kV. The micrographs were taken with the Olympus Keep view CCD Camera attached to the microscope.

**Quantitative Real time PCR:**

Total RNA from the xenografts were extracted using Trizol reagent strictly following manufacturer's instruction. The total RNA was quantified using nanospectrophotometer. Approximately 1µg of total RNA was subjected to cDNA synthesis using high capacity cDNA reverse transcription kit (Thermofisher scientific). Real-time PCR was performed on Real-Time PCR Detection System (AB 7500, Applied Biosystems, USA) using SYBR Green chemistry. Assays were done in 10 µl reaction consisting of 2X SYBR mastermix (Qiagen), 5 picomoles of forward and reverse primers each and 100 ng of cDNA. Milli Q water was used for negative controls. The protocol consisted of 95°C for 10 minutes, followed by 40 cycles of amplification of 95°C for 10 seconds and 60°C for 1 minute. GAPDH served as the endogenous control. The results were quantified by delta-delta Ct method and expressed as log2 fold change normalized to control group.

**Analysis of human apoptosis array:**

Human Apoptosis Array (R&D systems, Minneapolis, USA) was performed on the control, GNPs-1 and GNPs-2 groups strictly following the manufacturer´s instructions. This array allows

a parallel determination of 35 apoptosis related proteins on the tumor lysates. Tumor lysates from the xenografts were prepared using the lysis buffer provided in the kit. Tumor lysate (400 μg) was used for the analysis of apoptosis. The array was processed as per manufacturer's instructions. Briefly, 2.0 ml of blocking buffer (array buffer 1) was added into each well to block the membrane followed by 1h incubation on a rocking platform. Tumor lysate (400 μg in 250μl) from each sample was added, and the array was incubated at 4°C overnight in dark on a rocking platform. After incubation, the array was washed thrice with 20 mL of 1X wash buffer for every 10 minutes. Reconstituted antibody cocktail (15 μl in 1.5 mL of 1X dilution buffer ( array buffer 2/3)) was added to the array. The array was washed thrice. The excess wash buffer was allowed to drain from the array. The Streptavidin-HRP (2ml) was added to each array and incubated for 30 minutes. The array was washed and incubated with Chemiluminescence reagent (supplied in the kit) for a minute. The image was captured in a Chemi Documentation system (Biorad). The image files were processed using Imagelab software (Biorad). The pixel intensities were calculated using volume function by selecting the spots in the array. The automated local background selection was used to derive the mean intensity of the spots. The arrays were normalized to reference spot to adjust for inter-array variations. The results were statistically analyzed using One way ANOVA with Tukey's multiple comparison test to compare between the groups. A p value $<0.05$ was considered to be significant.

**Supplementary Figure Legends:**

***Fig S1: Flowchart of multivariate data analysis following FPA-FTIR.*** The spectra obtained post FTIR spectroscopy were pre-processed and subjected to multivariate data analysis by HCA and PCA.

***Fig S2: Characterization of GNP conjugates.*** The GNPs-1 and GNPs-2 conjugates were characterized by FTIR and TEM. The FTIR analysis spectra were similar between both the conjugates showing six significant peaks and confirmed proper conjugation of HDM2 peptide to respective GNPs (A). The TEM images of the conjugates internalized in the Y79 cells confirmed the morphology and size of the conjugates in the range of 15-20 nm and also suggested that they were stable in the cellular environment (B).

***Fig S3: Biochemical and hematological parameters f of dose-tolerance and non-toxicity of GNP conjugates.*** The liver, kidney function tests and differential leukocyte counts (DLC) were carried out for the analysis of dose-tolerance and toxic nature of the treatment. The levels of alanine aminotransferase (ALT) (p=0.6129) (A) and aspartate aminotransferase (AST) (p=0.9561) (B) as indicators of liver function and levels of Creatinine (p=0.1277) (C) and blood urea nitrogen (BUN) (p=0.0606) (D) as indicators of kidney function did not show significant difference between the groups confirming the dose-tolerance and non- toxic nature of the treatment to the vital organs**.** The DLC revealed no significant changes in the percentage of eosinophils (p=0.3677); monocytes (p=0.2211); lymphocytes (p=0.4192) and neutrophils (p=0.5630), compared to the controls (E) suggesting the absence of bone marrow suppression.

***Fig S4: Histopathological analysis of vital organs from control and treated mice at the end of the treatment.*** The histopathological analysis of liver, lung, spleen, heart and kidney in all the groups appeared normal, substantiating the dose tolerance and non-toxic nature of the treatments.

Abbreviations: Liver-PT- portal triad; CV- central vein; Hp- Hepatocytes; Lung-A-Alveoli; Br-Bronchiole; BV- Blood vessel; Spleen-WP- White Pulp; RP-Red pulp; C-cords; Heart-All the fields show evenly arranged striated, cardiac muscles with a few RBCs in between the muscle fibers; Kidney- T- Tubules; G- Glomeruli; BV- Blood vessel. Magnification – 200x.

***Fig S5:* Mechanism involved in the response to the treatment with GNP conjugates in the xenograft models.**

The real-time PCR and apoptotic array analysis were carried out to find the mechanism behind the response to the different treatments. The real time PCR analysis revealed up-regulation of p53 in GNPs-1 ($p<0.0001$; One way ANOVA) with concurrent down-regulation in the GNPs-2 ($p<0.0001$) compared to controls suggesting p53 dependent and p53 independent pathways, respectively (A). The transcript levels of HDM2 were found to be significantly down-regulated in both the treated groups compared to control ($p<0.0001$; One way ANOVA) (B). The apoptotic array analysis on the three groups showed differences in 17 out of 35 proteins analyzed (C). The results show that p53 dependent apoptotic pathway was engaged by GNPs-1 treatment and p53 independent and Bcl2 directed autophagic cell death was engaged by GNPs-2 to bring about the therapeutic response (C). In addition, downregulation of HIF 1 alpha in the absence of p53 activation may lead to tumor growth suppression (C). The increased levels of Hsp60 and survivin are suggestive of the non-engagement of the HDM2 peptide based therapy in controlling this pathway that may have reduced the efficacy of the current treatment (C).
